# Intense photoluminescence in CaTiO_3_:Sm^3+^ phosphors, effect of co-doping singly, doubly and triply ionized elements and their applications in LEDs

**DOI:** 10.1039/d3ra04468h

**Published:** 2023-07-26

**Authors:** Priti Singh, Sumit Modanwal, Hirdyesh Mishra, Shyam Bahadur Rai

**Affiliations:** a Laser and Spectroscopy Laboratory, Department of Physics, Institute of Science, Banaras Hindu University Varanasi 221005 India sbrai49@yahoo.co.in; b Physics Section, Mahila Maha Vidyalaya, Department of Physics, Banaras Hindu University Varanasi 221005 India hmishra@bhu.ac.in

## Abstract

In this work, Sm^3+^-doped and Sm^3+^/Li^+^/K^+^/Mg^2+^/Ba^2+^/Gd^3+^/Bi^3+^ co-doped CaTiO_3_ phosphors were synthesized by a solid-state reaction method at 1473 K. The phase of phosphors was identified to be orthorhombic with space group *Pnma* (62) by XRD measurements. The morphological properties of the prepared samples were studied by SEM measurements. The average crystallite and particle sizes were found to increase in the presence of modifiers and they follow the trend Li^+^ > Mg^2+^ > Gd^3+^ > K^+^ > Bi^3+^ > Ba^2+^. EDX measurements were used to verify the presence of Ca, Ti, O, Sm, K, Mg, Ba, Gd and Bi atoms in the prepared phosphor samples. The Sm^3+^ ion shows emission peaks at 564, 599 and 646 nm due to ^4^G_5/2_ → ^6^H_5/2_, ^6^H_7/2_ and ^6^H_9/2_ transitions upon 407 nm excitation, among which the peak situated at 599 nm has maximum emission intensity. Concentration quenching was observed above 2 mol% of Sm^3+^ ions in this host. However, the emission intensity of Sm^3+^ peaks can be enhanced using different modifier (Li^+^/K^+^/Mg^2+^/Ba^2+^/Gd^3+^/Bi^3+^) ions. It was found that the size (ionic radii) and charge compensation of the ion together play a dominant role. The enhancement is more after co-doping with smaller radius ions (Li^+^, Mg^2+^ and Gd^3+^), among which Li^+^ shows the largest enhancement. This is because ions of smaller size will be able to go closer to the activator ion and the charge imbalance causes a larger field. The CIE color coordinates, correlated color temperature (CCT) and color purity of the phosphors were calculated and show orange-red color emissions with a maximum color purity of ∼93% in the case of CaTiO_3_:2Sm^3+^/1.0Li^+^ phosphor. The lifetime value is increased in the presence of these ions. It is again maximum for the Li^+^ co-doped CaTiO_3_:2Sm^3+^ phosphor sample. Thus, the prepared phosphor samples are suitable sources for orange-red light.

## Abstract 2

Enhancement in emission intensity of Sm^3+^ ion in the presence of smaller and larger ionic radii ions and the emitted orange-red color used in LED applications.

## Graphical abstract

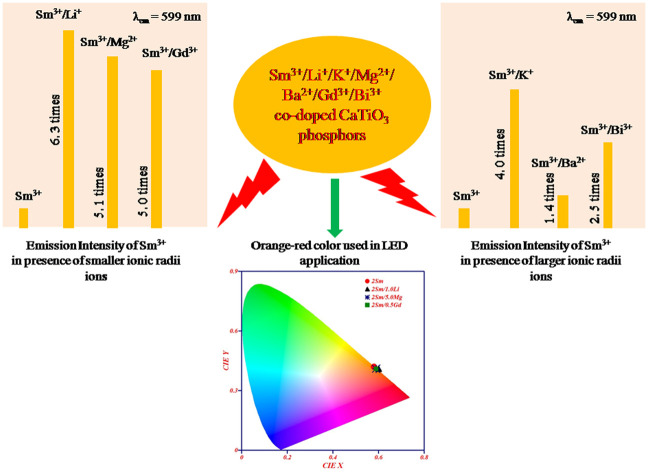

## 1. Introduction

Rare earth-doped perovskite phosphor materials are chemically and thermally stable and give intense photoluminescence at suitable excitation wavelengths.^1–3^ These materials are used for various applications such as color tunable devices, display devices, light-emitting devices, plasma display panels, temperature sensors, optical heaters, bio-imaging devices, plant growth and solar cells.^4–10^ The rare earth ions possess ladder-like energy levels due to which they show multi-modal behaviours such as upconversion (UC), downshifting (DS) and quantum cutting (QC) depending on different excitation detection techniques.^11–14^

Downshifting is a Stokes emission process, in which a high energy photon is converted into a low energy photon *via* different relaxation processes. Among the rare earth ions, the Sm^3+^ ion emits orange-red emissions due to ^4^G_5/2_ → ^6^H_*j*_ (*j* = 5/2, 7/2 and 9/2) transitions under n-UV excitation.^15–17^ Ha *et al.* studied the structure and photoluminescence properties of the Sm^3+^-doped CaTiO_3_ phosphor and observed intense orange-red emissions due to the ^4^G_5/2_ → ^6^H_*j*_ transition under 408 nm excitation.^18^ Shivaram *et al.* synthesized Sm^3+^-doped CaTiO_3_ by a low-temperature solution combustion method and reported an intense emission peak at 601 nm due to the ^4^G_5/2_ → ^6^H_7/2_ transition under 407 nm excitation.^19^ Generally, the orange/red-emitting phosphors show poor luminescence efficiency as compared to green, yellow and blue-emitting phosphors and need to be improved using different sensitizer/modifier ions.^20–22^ The emission intensity of activator ions may be enhanced in two ways: the first one is by co-doping with surface modifier ions such as Li^+^, Na^+^, Sr^2+^, Ca^2+^, Ba^2+^, Mg^2+^, Bi^3+^, Gd^3+^ and the second one is *via* energy transfer from the sensitizer to activator ions.^23–28^ Cao *et al.* observed an enhancement in the emission intensity of Sm^3+^-doped CaTiO_3_ phosphors *via* the addition of Na^+^ and H_3_BO_3_.^29^ Shanbhag *et al.* have reported the photoluminescence properties of CaTiO_3_:Sm^3+^/Li^+^.^30^ Pamuluri *et al.* have tried to enhance the photoluminescence properties of Sm^3+^ by energy transfer from Dy^3+^ to Sm^3+^ in Dy^3+^/Sm^3+^ co-doped Lu_3_Ga_5_O_12_ nano-garnets.^31^ Zhu *et al.* also observed an enhancement in the emission intensity of Sm^3+^ ions *via* energy transfer from Tb^3+^ to Sm^3+^ in Na_3_Bi(PO)_4_.^32^ Dhananjaya *et al.* have reported the PL properties of Eu^3+^-doped Gd_2_O_3_ phosphors in the presence of alkali ions (M^+^ = Li^+^, Na^+^ and K^+^).^33^ They have observed that the PL intensity of phosphors is increased in the presence of alkali ions, which is due to the modification in the local crystal field around the activator ion. In our previous work, we have observed an enhancement in the emission intensity of Eu^3+^-doped CaTiO_3_ phosphors in the presence of alkali ions (Li^+^, Na^+^ and K^+^).^34^ The enhancement in the emission intensity in the presence of alkali ions is due to the modification in crystal field around the activator ion, which increases the average crystallite and particle size. Singh *et al.* have studied the photoluminescence properties of Eu^3+^-doped MSiO_3_ in the presence of alkaline earth ions (M = Mg^2+^, Ca^2+^, Sr^2+^ and Ba^2+^) and found an intense emission of Eu^3+^ bands due to the modification in the crystal field around the activator ion.^35^ Wang *et al.* reported the enhancement in the luminescence properties of SrIn_2_O_4_:Eu^3+^ phosphors in the presence of Gd^3+^ ions.^36^ It is clear from these examples that ions of smaller as well as larger size with single, double and triple ionization state separately have been used to enhance the emission intensity of different rare earth ions in different hosts. All these ions modify the crystal field around the activator ions due to which enhancement in emission intensity is observed. However, from all these studies, it is not clear whether the ionization state or size of the ion or both plays a dominant role in enhancing the photoluminescence emission intensity of the activator ions. Therefore, it will be interesting to study these in detail. The idea is to see whether the ionization state or size of ions and charge compensation behaviors are more effective in enhancing the emission intensity of activators.

In this work, we studied the structural and optical behaviors of Sm^3+^-doped CaTiO_3_ phosphors in more detail in the presence of different types of surface modifier ions like singly, doubly and triply ionized elements with smaller and larger ionic radii in a single platform and tried to realize which one is more effective. The Sm^3+^-doped CaTiO_3_ and Li^+^/K^+^/Mg^2+^/Ba^2+^/Gd^3+^/Bi^3+^ co-doped CaTiO_3_:2Sm^3+^ phosphor materials were prepared by a solid-state reaction method at 1473 K. The XRD measurements showed that phosphor materials have an orthorhombic phase with the *Pnma* (62) space group. The SEM measurements were carried out to know the effect of different modifier ions on the particle size of the prepared samples. The photoluminescence excitation and emission spectra of the Sm^3+^-doped CaTiO_3_ phosphors were studied by taking *λ*_em_ = 599 nm and *λ*_ex_ = 407 nm, respectively. The concentration of Sm^3+^ was optimized for optimum emission. To improve the emission intensity further, Li^+^/K^+^/Mg^2+^/Ba^2+^/Gd^3+^/Bi^3+^ ions (smaller and larger ionic radii of singly, doubly and triply ionized elements) were co-doped in the CaTiO_3_:2Sm^3+^ phosphor and their concentrations were varied to obtain the optimum emission. It was found that the enhancement is more in the case of smaller radius ions irrespective of their ionization state and it is optimum for Li^+^ ions. We also calculated the CIE, CCT and color purity of the Sm^3+^ ions in the presence of Li^+^/K^+^/Mg^2+^/Ba^2+^/Gd^3+^/Bi^3+^ ions. The value of color purity is larger in the presence of smaller radius (Li^+^/Mg^2+^/Gd^3+^) ions and they follow the trend Li^+^ > Mg^2+^ > Gd^3+^. The lifetime measurements were carried out for the ^4^G_5/2_ level of Sm^3+^ ions in the absence and presence of these modifier ions. It was found that the lifetime of the ^4^G_5/2_ level of Sm^3+^ ions increased in the presence of modifier ions and their order is *τ*_Li_ > *τ*_k_ ∼ *τ*_Gd_ > *τ*_Mg_ > *τ*_Bi_ > *τ*_Ba_.

## 2. Preparation of phosphor samples and characterization

### 2.1. Synthesis of materials

The phosphor samples were synthesized by a solid-state reaction technique at 1473 K. The starting materials were calcium carbonate (CaCO_3_, 99.9%), titanium dioxide (TiO_2_, 99.9%), samarium oxide (Sm_2_O_3_, 99.9%), lithium carbonate (Li_2_CO_3_, 99%), potassium carbonate (K_2_CO_3_, 99%) magnesium oxide (MgO, 97%), barium carbonate (BaCO_3_, 99.9%), gadolinium oxide (Gd_2_O_3_, 99.9%) and bismuth oxide (Bi_2_O_3_, 99.9%). Initially, a series of *c*Sm^3+^ (*c* = 1.0, 1.5, 2.0, 2.5, 3.0 and 5.0 mol%) doped CaTiO_3_ phosphor samples were synthesized to find the Sm^3+^ concentration for maximum photoluminescence. The Sm^3+^ ion was found to give optimum emission at 2 mol%. Further, in order to enhance the PL intensity of Sm^3+^, *x*Li^+^ (where *x* = 0.5, 1.0 and 3.0 mol%), *x*K^+^ (where *x* = 1.0, 3.0, 5.0 and 7.0 mol%), *y*Mg^2+^ (where *y* = 1.0, 3.0, 5.0 and 10 mol%), *y*Ba^2+^ (where *y* = 3.0, 5.0 and 10 mol%), *z*Gd^3+^ (where *z* = 0.1, 0.5 and 0.7 mol%) and *z*Bi^3+^ (where *z* = 3.0, 5.0 and 10 mol%) were co-doped in CaTiO_3_:2 mol% Sm^3+^ phosphors separately to get the optimum emission of Sm^3+^ ions in the presence of these ions.

The weighed materials were carefully mixed for one hour in an agate mortar with acetone as a mixing medium. The final mixtures were heated at 1473 K for 4 hours in a programmable electric furnace. The phosphor samples thus obtained were further crushed in an agate mortar to obtain a fine powder for further characterizations.

### 2.2. Instrumentation

The phase identification of phosphor materials was carried out by XRD measurements using CuK_α_ radiation (*λ* = 0.15406 nm) with MiniFlex600 (Rigaku, Japan). The morphology of the materials was studied by SEM using a Zeiss, Evo 18 Research system. The Fourier transform infrared measurements were done to know the phonon frequency of phosphor samples using a PerkinElmer I-Frontier system in the 400–3000 cm^−1^ region. The photoluminescence excitation and emission spectra of the samples were recorded using a Fluorolog-3 spectrophotometer with a 450 W xenon lamp source (Horiba Jobin Yvon). We also measured the lifetime of the ^4^G_5/2_ level of Sm^3+^ ions in the absence and presence of Li^+^/K^+^/Mg^2+^/Ba^2+^/Gd^3+^/Bi^3+^ ions using a 25 W pulsed xenon lamp attached with the same unit (only corresponding to optimized samples).

## 3. Results and discussion

### 3.1. Structural characterization

#### 3.1.1. XRD measurements

**Fig. 1:**
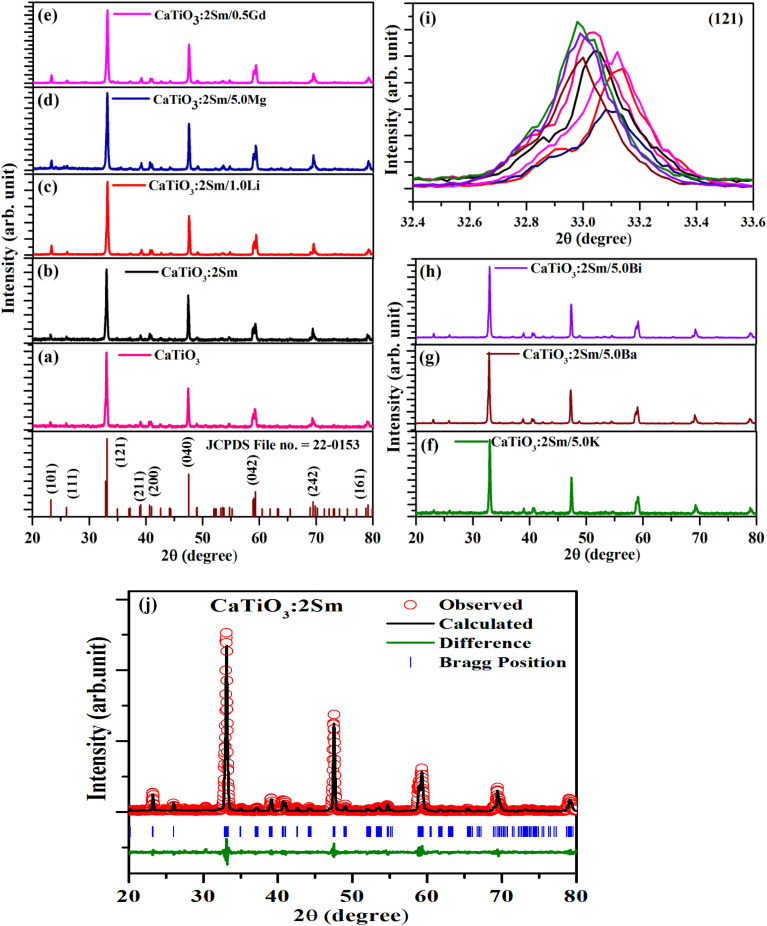
XRD patterns of (a) CaTiO_3_, (b) CaTiO_3_:2Sm^3+^, (c) CaTiO_3_:2Sm^3+^/1.0Li^+^, (d) CaTiO_3_:2Sm^3+^/5.0Mg^2+^, (e) CaTiO_3_:2Sm^3+^/0.5Gd^3+^, (f) CaTiO_3_:2Sm^3+^/5.0K^+^, (g) CaTiO_3_:2Sm^3+^/5.0Ba^2+^, (h) CaTiO_3_:2Sm^3+^/5.0Bi^3+^ phosphors and (i) shifting in the (121) peak in the range of 32.4 to 33.6°, 2*θ* angle. (j) Rietveld refinements for the CaTiO_3_:2Sm^3+^ phosphor.

The powder X-ray diffraction patterns of CaTiO_3_, CaTiO_3_:2Sm^3+^, CaTiO_3_:2Sm^3+^/1.0Li^+^, CaTiO_3_:2Sm^3+^/5.0Mg^2+^, CaTiO_3_:2Sm^3+^/0.5Gd^3+^, CaTiO_3_:2Sm^3+^/5.0K^+^, CaTiO_3_:2Sm^3+^/5.0Ba^2+^ and CaTiO_3_:2Sm^3+^/5.0Bi^3+^ phosphor samples were monitored in the range of 20–80°, 2*θ* angles, and they are shown in Fig. 1(a)–(h). The phase of the phosphors were identified to be orthorhombic with space group *Pnma* (62). There were no extra peaks due to substituent ions; however, the XRD peaks show shifts due to substitution. The sharp diffraction peaks also confirm the crystalline nature of the prepared samples. When the lower radius ions (Li^+^/Mg^2+^/Gd^3+^) are co-doped in the CaTiO_3_:2Sm^3+^ phosphor at Ca^2+^ sites, a shifting of peaks in the higher angle side was observed in the diffraction peaks. However, on doping of higher radius ions (K^+^/Ba^2+^/Bi^3+^) at the Ca^2+^ site, a shifting of peaks in the lower angle side was observed in the diffraction peaks. Upon doping of Sm^3+^ (0.095) at Ca^2+^ sites, the diffraction peaks are shifted slightly towards a higher 2*θ* angle side. These shifts observed in different cases are shown in Fig. 1(i) for intense peak (121) in the range of 32.4 to 33.6°, 2*θ* angle.

The ionic radii of Li^+^, Mg^2+^ and Gd^3+^ are 0.076, 0.072 and 0.093 nm, while the ionic radii of Ca^2+^ is 0.100 nm. Therefore, on substitution of Li^+^, Mg^2+^ and Gd^3+^ ions at the Ca^2+^ site, the crystal lattice shrinks due to which the XRD peaks are shifted towards a higher 2*θ* angle side. Wu *et al.* have also observed the shift in peaks to a higher 2*θ* angle side on co-doping of Li^+^ (smaller ionic radii) at the Ca^2+^ site in CaTiO_3_:Eu^3+^ phosphors.^37^ However, the ionic radii of K^+^, Ba^2+^ and Bi^3+^ are 0.138, 0.135 and 0.103 nm, which are larger than that of Ca^2+^ ionic radii. Therefore, on substitution of K^+^, Ba^2+^ and Bi^3+^ at the place of Ca^2+^ the crystal lattice expands due to which the XRD peaks are shifted towards a lower 2*θ* angle side.

The Rietveld refinements for the CaTiO_3_:2Sm^3+^ phosphor sample were carried out, and they are shown in Fig. 1(j). The lattice parameters are *a* = 5.4420, *b* = 7.6463, and *c* = 5.3840, with volume *V* = 224.0342, and conventional Rietveld parameters were *R*_p_ = 14.2, *R*_wp_ = 23.7, *R*_exp_ = 18.7, and *χ*^2^ = 1.59.^38^

The average crystallite size (*D*) of the phosphor samples was calculated using the Debye–Scherrer (D–S) equation:^39^1
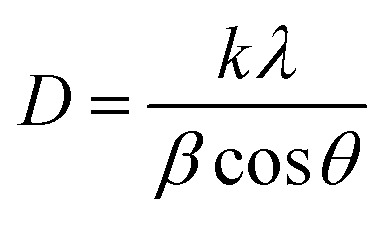
where *D* is the average crystallite size, *k* is the shape factor (0.89), *λ* is the X-ray wavelength, *β* is the full width at half maximum (FWHM) and *θ* is the diffraction angle. The average crystallite size values were found to be 30.0, 31.02, 35.51, 33.40, 33.05, 32.80, 31.50 and 32.20, nm for the CaTiO_3_, CaTiO_3_:2Sm^3+^, CaTiO_3_:2Sm^3+^/1.0Li^+^, CaTiO_3_:2Sm^3+^/5.0Mg^2+^, CaTiO_3_:2Sm^3+^/0.5Gd^3+^, CaTiO_3_:2Sm^3+^/5.0K^+^, CaTiO_3_:2Sm^3+^/5.0Ba^2+^ and CaTiO_3_:2Sm^3+^/5.0Bi^3+^ phosphor samples, respectively. This shows that the crystallite size corresponding to Li^+^, Mg^2+^, and Gd^3+^ are slightly larger than that of K^+^, Ba^2+^, Bi^3+^. The dislocation density was used to know the optical efficiency of the phosphor materials. The dislocation density was calculated from the crystallite size using the following relation:^34^2
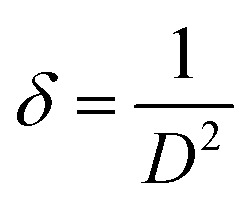


The dislocation density values for CaTiO_3_, CaTiO_3_:2Sm^3+^, CaTiO_3_:2Sm^3+^/1.0Li^+^, CaTiO_3_:2Sm^3+^/5.0Mg^2+^, CaTiO_3_:2Sm^3+^/0.5Gd^3+^, CaTiO_3_:2Sm^3+^/5.0K^+^, CaTiO_3_:2Sm^3+^/5.0Ba^2+^ and CaTiO_3_:2Sm^3+^/5.0Bi^3+^ phosphor samples were found to be 11.1 × 10^−4^, 10.3 × 10^−4^, 7.9 × 10^−4^, 8.9 × 10^−4^, 9.1 × 10^−4^, 9.3 × 10^−4^, 10.0 × 10^−4^ and 9.6 × 10^−4^ nm^−2^, respectively. Thus, the dislocation density values were found to decrease and the average crystallite size increase in case of CaTiO_3_:2Sm^3+^/1.0Li^+^, CaTiO_3_:2Sm^3+^/5.0Mg^2+^, CaTiO_3_:2Sm^3+^/0.5Gd^3+^, CaTiO_3_:2Sm^3+^/5.0K^+^, CaTiO_3_:2Sm^3+^/5.0Ba^2+^ and CaTiO_3_:2Sm^3+^/5.0Bi^3+^ phosphors. Therefore, the PL intensity of Sm^3+^ ions is expected to enhance in the presence of Li^+^/K^+^/Mg^2+^/Ba^2+^/Gd^3+^/Bi^3+^ ions due to the increase in average crystallite size.

#### 3.1.2. SEM and EDX measurements

**Fig. 2:**
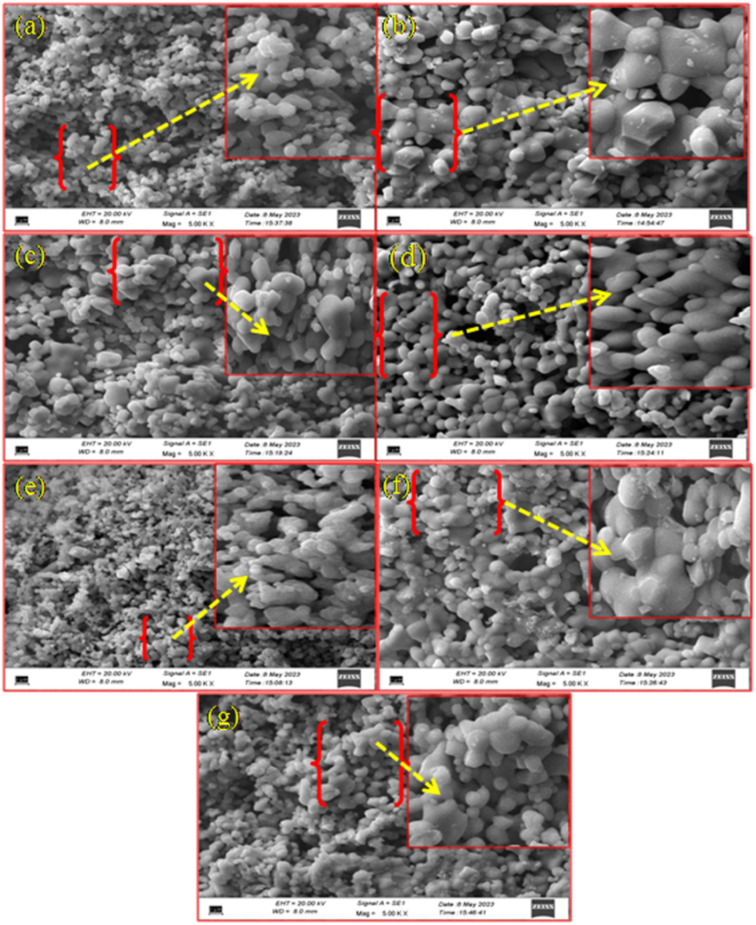
SEM images of (a) CaTiO_3_:2Sm^3+^, (b) CaTiO_3_:2Sm^3+^/1.0Li^+^, (c) CaTiO_3_:2Sm^3+^/5.0K^+^, (d) CaTiO_3_:2Sm^3+^/5.0Mg^2+^, (e) CaTiO_3_:2Sm^3+^/5.0Ba^2+^, (f) CaTiO_3_:2Sm^3+^/0.5Gd^3+^, and (g) CaTiO_3_:2Sm^3+^/5.0Bi^3+^ phosphor samples.

**Fig. 3:**
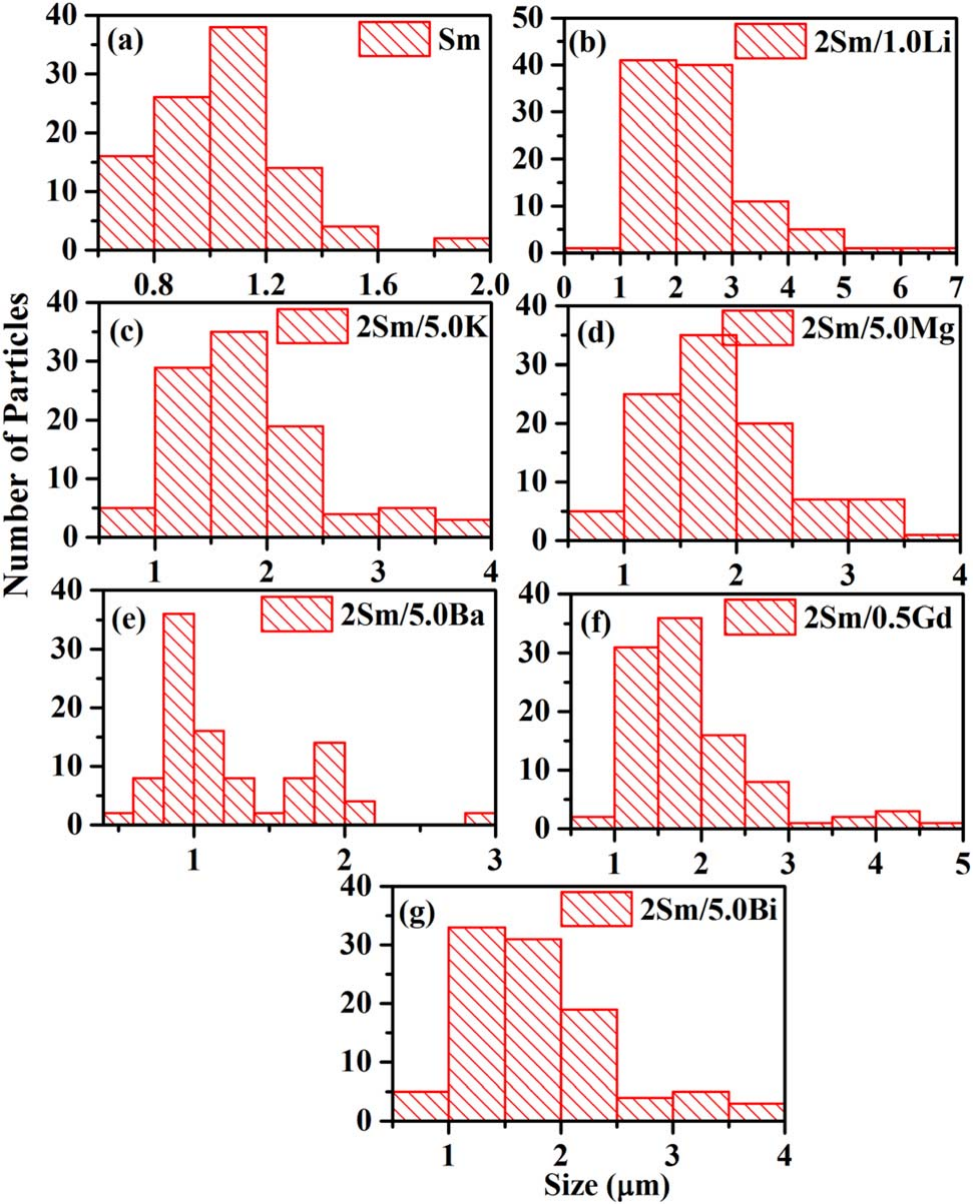
Particle size distribution of (a) CaTiO_3_:2Sm^3+^, (b) CaTiO_3_:2Sm^3+^/1.0Li^+^, (c) CaTiO_3_:2Sm^3+^/5.0K^+^, (d) CaTiO_3_:2Sm^3+^/5.0Mg^2+^, (e) CaTiO_3_:2Sm^3+^/5.0Ba^2+^, (f) CaTiO_3_:2Sm^3+^/0.5Gd^3+^ and (g) CaTiO_3_:2Sm^3+^/5.0Bi^3+^ phosphor samples.

The surface morphology of the prepared phosphor samples was studied by SEM measurements. Fig. 2(a)–(g) show the SEM images of CaTiO_3_:2Sm^3+^, CaTiO_3_:2Sm^3+^/1.0Li^+^, CaTiO_3_:2Sm^3+^/5.0K^+^, CaTiO_3_:2Sm^3+^/5.0Mg^2+^, CaTiO_3_:2Sm^3+^/5.0Ba^2+^, CaTiO_3_:2Sm^3+^/0.5Gd^3+^ and CaTiO_3_:2Sm^3+^/5.0Bi^3+^ phosphor samples, respectively. However, the enlarged picture of a small part of these images in all the cases is shown in the right top corner. In almost all cases, particles are slightly agglomerated to each other and nearly spherical or slightly elongated in shape. It is clear from the enlarged part of the images that the particle sizes are larger in the cases of Li^+^, Mg^2+^ and Gd^3+^ compared to K^+^, Ba^2+^ and Bi^3+^ ions. The average particle size of the prepared phosphors was calculated by the SEM images from the histogram using the ImageJ software and these values were found to be 1.02, 2.19, 1.69, 1.77, 1.24, 1.73 and 1.66 μm for CaTiO_3_:2Sm^3+^, CaTiO_3_:2Sm^3+^/1.0Li^+^, CaTiO_3_:2Sm^3+^/5.0K^+^, CaTiO_3_:2Sm^3+^/5.0Mg^2+^, CaTiO_3_:2Sm^3+^/5.0Ba^2+^, CaTiO_3_:2Sm^3+^/0.5Gd^3+^ and CaTiO_3_:2Sm^3+^/5.0Bi^3+^ phosphor samples, respectively [see Fig. 3(a)–(g)]. Thus, the average particles size was found to increase in the presence of all the ions and it is maximum for CaTiO_3_:2Sm^3+^/1.0Li^+^ phosphors. The Li^+^/K^+^/Mg^2+^/Ba^2+^/Gd^3+^/Bi^3+^ ion actually acts as a surface modifier, which modifies the local crystal field around the Sm^3+^ ions in the CaTiO_3_ matrix, and therefore, improves the emission intensity in the presence of these modifier ions. It is found maximum in the case of Li^+^ ions.

**Fig. 4:**
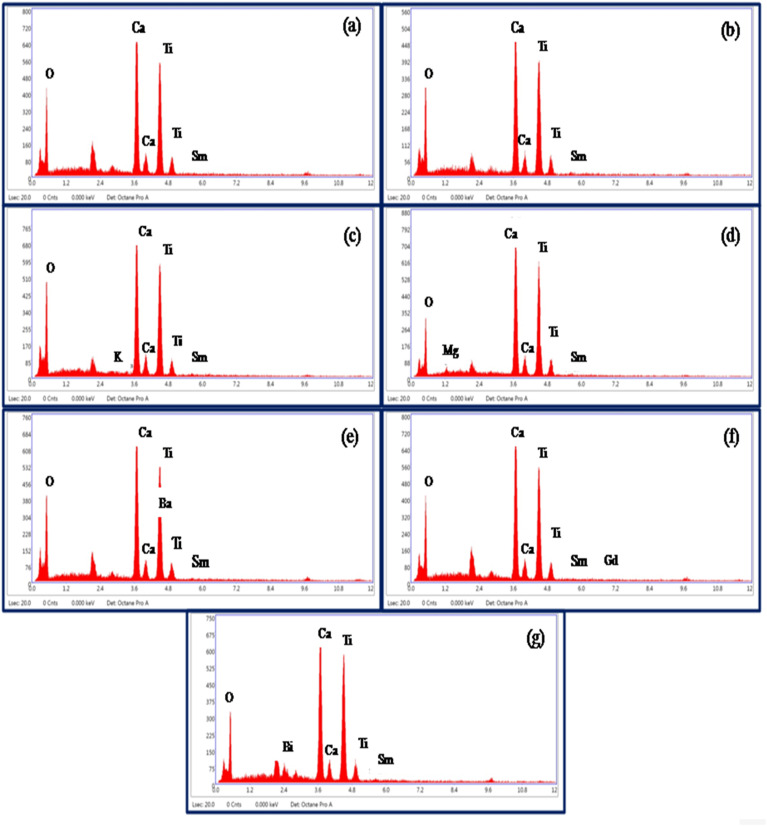
EDX spectra of (a) CaTiO_3_:2Sm^3+^, (b) CaTiO_3_:2Sm^3+^/1.0Li^+^, (c) CaTiO_3_:2Sm^3+^/5.0K^+^, (d) CaTiO_3_:2Sm^3+^/5.0Mg^2+^, (e) CaTiO_3_:2Sm^3+^/5.0Ba^2+^, (f) CaTiO_3_:2Sm^3+^/0.5Gd^3+^ and (g) CaTiO_3_:2Sm^3+^/5.0Bi^3+^ phosphor samples.

Energy-dispersive X-ray spectroscopic (EDX) measurements were used to know the elements present in the prepared phosphor samples. The EDX patterns of CaTiO_3_:2Sm^3+^, CaTiO_3_:2Sm^3+^/1.0Li^+^, CaTiO_3_:2Sm^3+^/5.0K^+^, CaTiO_3_:2Sm^3+^/5.0Mg^2+^, CaTiO_3_:2Sm^3+^/5.0Ba^2+^, CaTiO_3_:2Sm^3+^/0.5Gd^3+^ and CaTiO_3_:2Sm^3+^/5.0Bi^3+^ phosphor samples are shown in Fig. 4(a)–(g). The spectra show the presence of Ca, Ti, O, Sm, K, Mg, Ba, Gd and Bi elements in the prepared phosphor samples except the Li element. Because of its very light nature, it was ionized in the SEM chamber during interaction with the incident beam, and therefore, it could not be seen in the spectrum.^34^ This confirmed that all the elements which were used in the sample preparation are present in the prepared samples.

### 3.2. Optical characterization

#### 3.2.1. FTIR studies

**Fig. 5:**
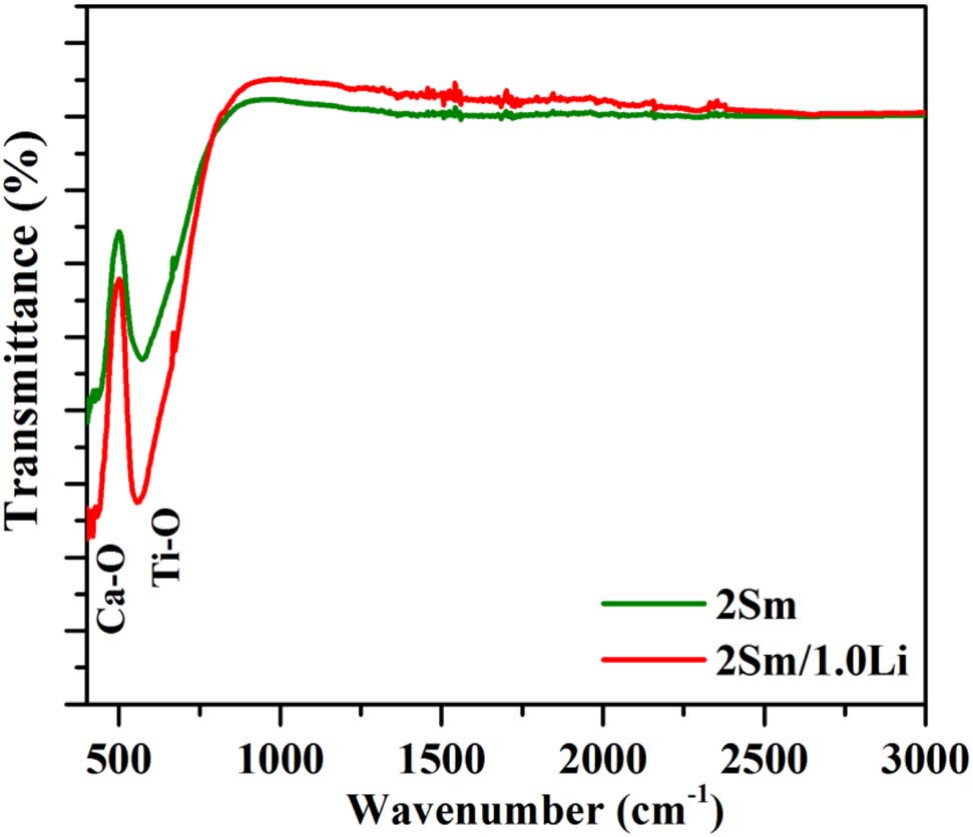
FTIR spectra of CaTiO_3_:2Sm^3+^ and CaTiO_3_:2Sm^3+^/1.0Li^+^ phosphors.

The Fourier transform infrared measurements were carried out to know the phonon frequencies (vibrational bands frequencies) of the phosphor samples. The FTIR spectra of CaTiO_3_:2Sm^3+^ and CaTiO_3_:2Sm^3+^/1.0Li^+^ phosphors in the spectral region 400–3000 cm^−1^ are shown in Fig. 5. The vibrational bands are observed at 430 and 545 cm^−1^ due to Ca–O and Ti–O groups.^34,39^

The spectra corresponding to modifier ions (Li^+^) are exactly identical except that there is a change in the intensity of the bands. The following conclusions could be drawn on the basis of these measurements. First, the phonon frequency of the phosphor materials is low, and hence, the non radiative relaxation process in this case will be poor. This means that the radiative emission in this case is expected to be high. Second, the vibrational frequency of the bands remains unchanged upon co-doping of the modifier. Only their intensity is affected due to scattering of photons with the modifier ion.

#### 3.2.2. Photoluminescence excitation spectrum and emission spectra of CaTiO_3_:*c*Sm^3+^ phosphors

**Fig. 6:**
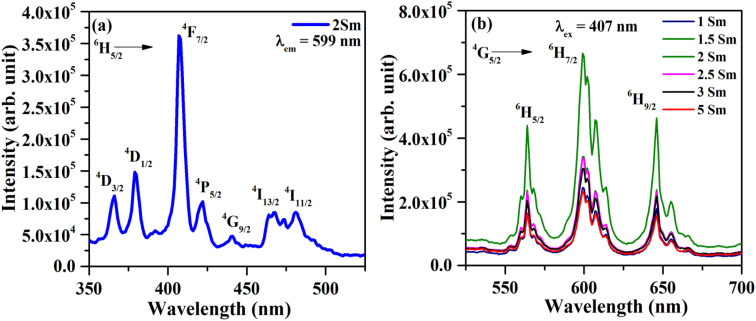
(a) Photoluminescence excitation (PLE) spectrum of the CaTiO_3_:2Sm^3+^ phosphor sample with *λ*_em_ = 599 nm and (b) photoluminescence emission (PL) spectra of CaTiO_3_:*c*Sm^3+^ (where *c* = 1.0, 1.5, 2.0, 2.5, 3.0 and 5.0 mol%) phosphor samples with *λ*_ex_ = 407 nm.

The photoluminescence excitation spectrum (PLE) of CaTiO_3_:2Sm^3+^ phosphor sample was monitored with *λ*_em_ = 599 nm in the 350–525 nm region, as shown in Fig. 6(a). The spectrum of CaTiO_3_:2Sm^3+^ phosphor contained a number of peaks, situated at 365, 380, 407, 422, 440, 467 and 481 nm due to ^6^H_5/2_ → ^4^D_3/2_, ^4^D_1/2_, ^4^F_7/2_, ^4^P_5/2_, ^4^G_9/2_, ^4^I_13/2_ and ^4^I_11/2_ transitions of the Sm^3+^ ion, respectively.^15–17^ The peak at 407 nm shows maximum intensity and lies in the region of the n-UV LED chip. Hence, this is important for LED applications.

The photoluminescence emission (PL) spectra of CaTiO_3_:*c*Sm^3+^ (where *c* = 1.0, 1.5, 2.0, 2.5, 3.0 and 5.0 mol%) phosphors with *λ*_ex_ = 407 nm in the spectral region 525–700 nm and they are depicted in Fig. 6(b). In the PL spectra, we observed three intense emission peaks at 564, 599, and 646 nm due to ^4^G_5/2_ → ^6^H_5/2_, ^6^H_7/2_ and ^6^H_9/2_ transitions of Sm^3+^ ions.^15–17^ The most intense peak is situated at 599 nm (^4^G_5/2_ → ^6^H_7/2_). The peak at 564 nm is due to the magnetic dipole transition. The most intense emission peak at 599 nm appears partially due to magnetic and partially due to forced electric dipole transitions. However, the peak at 646 nm is found due to electric dipole transition.

**Fig. 7:**
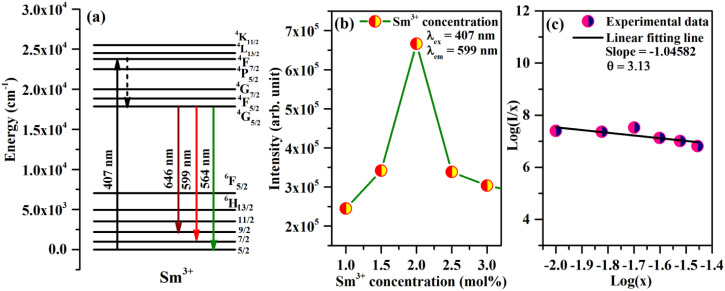
(a) Schematic energy level diagram of Sm^3+^ and the process involved in excitation and emission. (b) Variation of emission intensity with the concentration of Sm^3+^ ions. (c) Dual logarithmic plot between PL emission intensity per Sm^3+^ ion concentration [Log(*I*/*x*)] *versus* Sm^3+^ concentration [Log(*x*)].

The Sm^3+^ ions present in the ground state (^6^H_5/2_), are promoted to the ^4^F_7/2_ excited state by absorption of 407 nm photons. The Sm^3+^ ions from ^4^F_7/2_ excited state relax non-radiatively to the ^4^G_5/2_ excited state, which give multi-transition emission to different sublevels of the ground state, which lie in orange to red regions. The energy level diagram of Sm^3+^ ions is shown in Fig. 7(a). The photoluminescence emission intensity was found to increase with the concentrations of Sm^3+^ ions, and it was found optimum at 2 mol% in this host. The emission intensity was found to decrease for higher concentrations due to concentration quenching. The variation in emission intensity with different concentrations of Sm^3+^ ions is shown in Fig. 7(b). Two mechanisms are generally found to involve in concentration quenching. One is the exchange interaction and the other is the multipolar interaction. The two mechanisms depend on the critical distance between the activator ions. If the value of critical distance between the two ions is ≤5 Å, the concentration quenching would be due to exchange interaction. However, if it is ≥5 Å, it would be due to multipolar interaction.

The value of critical distance between Sm^3+^–Sm^3+^ ions could be calculated using the following equation:^34^3
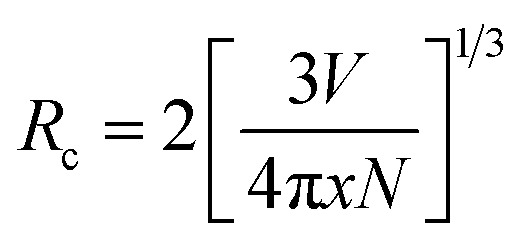
where *V* is the volume of unit cell, *x* is the optimum concentration of Sm^3+^ ions for which the emission intensity is maximum and *N* is the number of Ca^2+^ sites occupied by Sm^3+^ ions. In the present case, the value of *V* = 224.0342 Å^3^ and *x* = 0.02. The number of Ca sites, *i.e. N* = 4. This resulted in an *R*_c_ value of 17.44 Å, a value much larger than 5 Å. Hence, the concentration quenching in CaTiO_3_:*c*Sm^3+^ phosphors was found to be multipolar interaction.

The multipolar interactions can be recognized using the following formula:^34^4
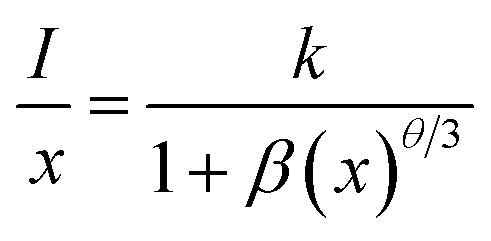
where *I* is the PL emission intensity, *x* is the Sm^3+^ ion concentration, *k* and *β* are the constants for a given host. The value of *θ* decides the actual nature of interaction between Sm^3+^ ions. Depending on the value of *θ* equal to 6, 8 or 10, the nature of interaction would be dipole–dipole (d–d), dipole–quadrupole (d–q) or quadrupole–quadrupole (q–q), respectively. A plot between Log(*I*/*x*) *versus* Log(*x*) for CaTiO_3_:*c*Sm^3+^ (where *c* = 1.0, 1.5, 2.0, 2.5, 3.0 and 5.0 mol%) phosphors with *λ*_ex_ = 407 nm and *λ*_em_ = 599 nm is shown in Fig. 7(c). The slope value of the curve was found by fitting the plot Log(*I*/*x*) *versus* Log(*x*). The observed slope value (equal to −*θ*/3) was found to be 1.04582, from which the value of *θ* ∼ 3.13, close to 3. If the value of *θ* is less than 6, the energy transfer is due to the interaction between the adjacent ions.

#### 3.2.3. Effect of co-doping of singly, doubly and triply ionized ions on the photoluminescence emission intensity of CaTiO_3_:2Sm^3+^ phosphor

**Fig. 8:**
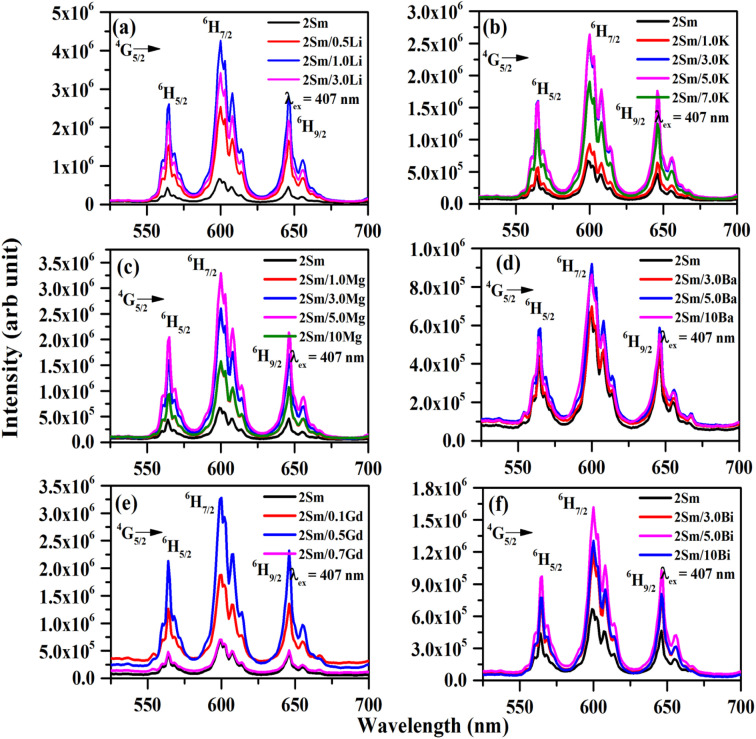
Photoluminescence emission (PL) spectra of (a) CaTiO_3_:2Sm^3+^/*x*Li^+^ (where *x* = 0, 0.5, 1.0 and 3.0 mol%), (b) CaTiO_3_:2Sm^3+^/*x*K^+^ (where *x* = 0, 1.0, 3.0, 5.0 and 7.0 mol%), (c) CaTiO_3_:2Sm^3+^/*y*Mg^2+^ (where *y* = 0, 1.0, 3.0, 5.0 and 10 mol%), (d) CaTiO_3_:2Sm^3+^/*y*Ba^2+^ (where *y* = 0, 3.0, 5.0 and 10 mol%), (e) CaTiO_3_:2Sm^3+^/*z*Gd^3+^ (where *z* = 0, 0.1, 0.5 and 0.7 mol%) and (f) CaTiO_3_:2Sm^3+^/*z*Bi^3+^ (where *z* = 0, 3.0, 5.0 and 10 mol%) phosphor samples with *λ*_ex_ = 407 nm.

The PL emission spectra of CaTiO_3_:2Sm^3+^/*x*Li^+^ (where *x* = 0.5, 1.0 and 3.0 mol%), *x*K^+^ (where *x* = 1.0, 3.0, 5.0 and 7.0 mol%), *y*Mg^2+^ (where *y* = 1.0, 3.0, 5.0 and 10 mol%), *y*Ba^2+^ (where *y* = 3.0, 5.0 and 10 mol%), *z*Gd^3+^ (where *z* = 0.1, 0.5 and 0.7 mol%) and *z*Bi^3+^ (where *z* = 3.0, 5.0 and 10 mol%) phosphors were monitored under excitation at 407 nm, and they are given in Fig. 8(a)–(f). The emission intensity is maximum for 1.0 mol% Li^+^, 5.0 mol% K^+^, 5.0 mol% Mg^2+^, 5.0 mol% Ba^2+^, 0.5 mol% Gd^3+^ and 5.0 mol% Bi^3+^ co-doped CaTiO_3_:2Sm^3+^ phosphors. As mentioned earlier, we selected two types of ions: one with a smaller size (Li^+^/Mg^2+^/Gd^3+^ with singly, doubly and triply ionized states) and the other with a larger size (K^+^/Ba^2+^/Bi^3+^ with singly, doubly and triply ionized states), and monitored the photoluminescence emission spectra under the same conditions. It was found that the emission wavelengths of the Sm^3+^ ion are the same. However, the photoluminescence emission intensity is increased in the presence of all these modifier ions. Moreover, it is larger in the case of ions with smaller ionic radii. The enhancement in the intensity of Sm^3+^ ions follow the trend *I*_Li^+^_ > *I*_Mg^2+^_ > *I*_Gd^3+^_ > *I*_K^+^_ > *I*_Bi^3+^_ > *I*_Ba^2+^_. Several researchers have used these ions to improve the emission intensity of rare earth ions in different host matrices.^23–27^ For example, Wu *et al.* have studied the photoluminescence properties of Eu^3+^/Li^+^ co-doped CaTiO_3_ phosphors.^37^ They found that the emission intensity of Eu^3+^ ions is increased in the presence of Li^+^ ions. They have explained it to be the result of the increase in average particle size and charge compensation. Our group have also observed an enhancement in the emission intensity of Tm^3+^/Yb^3+^ co-doped ZnWO_4_ phosphors in the presence of Mg^2+^ ions due to the modification in the local crystal field.^40^ Linga *et al.* have reported an enhancement in the photoluminescence intensity of (Ca_1−*x*−*y*_,Ln_*y*_)MoO_4_:*x*Eu^3+^ (Ln = Y and Gd) phosphors in the presence of Y^3+^ and Gd^3+^ ions.^41^

The emission intensity of CaTiO_3_:2Sm^3+^/1.0Li^+^, CaTiO_3_:2Sm^3+^/5.0K^+^, CaTiO_3_:2Sm^3+^/5.0Mg^2+^, CaTiO_3_:2Sm^3+^/5.0Ba^2+^, CaTiO_3_:2Sm^3+^/0.5Gd^3+^ and CaTiO_3_:2Sm^3+^/5.0Bi^3+^ phosphors was enhanced by 6.3, 4.0, 5.1, 1.4, 5.0 and 2.5 times (for 599 nm peak) compared to the CaTiO_3_:2Sm^3+^ phosphor.

These observations indicate that the ions with smaller size (ionic radii) are more effective in enhancing the emission intensity of Sm^3+^ ions. This is due to the reason that ions with a smaller ionic size will be able to reach closer to activator ions. The charge compensation will create a larger field to enhance the emission intensity. In the case of larger radius ions, whatever may be the ionization state (K^+^, Ba^2+^, and Bi^3+^), they will be struck out by the host atoms/ions before they reach closer to the activator ion (*i.e.* Sm^3+^) due to their larger size, and hence, the field created will be smaller. Thus, the ionic radii of Li^+^, Mg^2+^ and Gd^3+^ ions are 0.076, 0.072 and 0.093 nm, which are smaller than the Ca^2+^ ionic radii (0.100 nm) at which these ions are substituted, while the ionic radii of K^+^, Ba^2+^ and Bi^3+^ are 0.138, 0.135 and 0.103 nm, which are larger than the Ca^2+^ ionic radii. In all these, Li^+^, Mg^2+^ and Gd^3+^ ions produce larger enhancement and in that Li^+^ is the largest one. Another reason for the enhancement in emission intensity is if we compare the crystallite and particle size, they also follow the same trend, *i.e.* (Li^+^) > (Mg^2+^) > (Gd^3+^) > (K^+^) > (Bi^3+^) > (Ba^2+^). As larger the particle size, it contains a large number of activators which give large emission intensity. The increase in average crystallite and particle sizes in the presence of Li^+^/K^+^/Mg^2+^/Ba^2+^/Gd^3+^/Bi^3+^ ions improves the population of the ^4^G_5/2_ level. Therefore, the emission intensity is enhanced in the presence of Li^+^/K^+^/Mg^2+^/Ba^2+^/Gd^3+^/Bi^3+^ ions.^42^ The emission intensity is maximum in the case of CaTiO_3_:2Sm^3+^/1.0Li^+^ phosphor and it is due to its smaller ionic radii and used as a charge compensator as well as it has the largest average crystallite and particle size.

#### 3.2.4. Color coordinates, color purity and correlated color temperature calculations

**Fig. 9:**
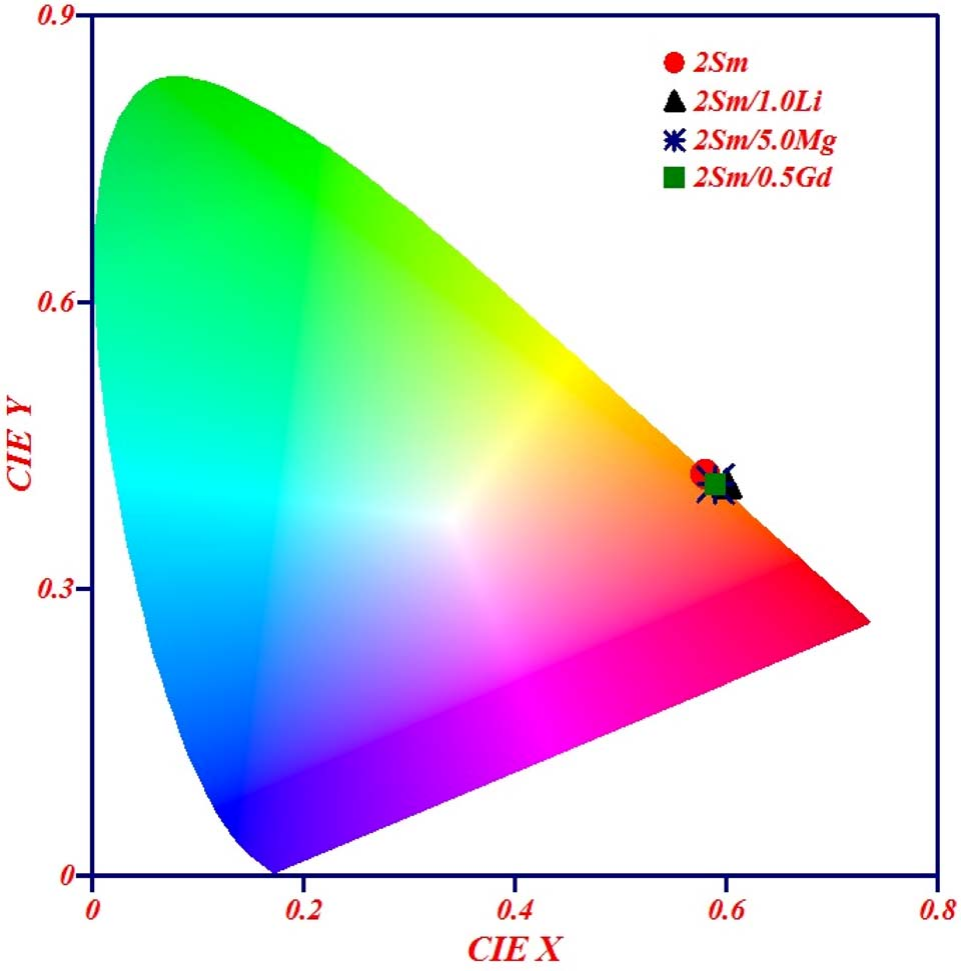
CIE diagram of CaTiO_3_:2Sm^3+^, CaTiO_3_:2Sm^3+^/1.0Li^+^, CaTiO_3_:2Sm^3+^/5.0Mg^2+^ and CaTiO_3_:2Sm^3+^/0.5Gd^3+^ phosphor samples upon 407 nm excitation.

The CIE (Commission Internationale de I'Eclairage) diagram is an excellent tool to verify the color emitted from the phosphors. The values of CIE coordinates were calculated for different samples, and they are shown in Fig. 9 for CaTiO_3_:2Sm^3+^ (0.58, 0.42), CaTiO_3_:2Sm^3+^/1.0Li^+^ (0.60, 0.41), CaTiO_3_:2Sm^3+^/5.0Mg^2+^ (0.59, 0.41), CaTiO_3_:2Sm^3+^/0.5Gd^3+^ (0.59, 0.41), CaTiO_3_:2Sm^3+^/5.0K^+^ (0.58, 0.42), CaTiO_3_:2Sm^3+^/5.0Ba^2+^ (0.58, 0.42), and CaTiO_3_:2Sm^3+^/5.0Bi^3+^ (0.58, 0.42), phosphors. The values of CIE coordinates are nearly the same in the presence of different ions and they lie in the orange red region.

The color purity of the phosphor materials is another important parameter to recognize phosphor as a good source of light for a particular color for solid-state lighting applications. The color purity of the light source can be calculated using the following relation:^43^5
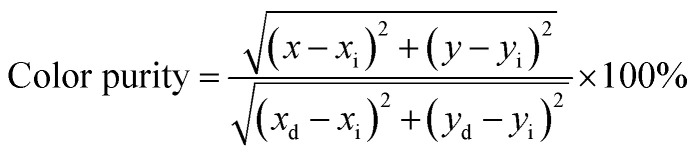
where (*x*, *y*) are the CIE coordinates of the phosphor, (*x*_d_, *y*_d_) are the CIE coordinates of the dominant wavelength and (*x*_i_, *y*_i_) are the CIE coordinates for the standard light source. The values of color purity in the present case were found to be 87.80% for CaTiO_3_:2Sm^3+^, CaTiO_3_:2Sm^3+^/5.0K^+^, CaTiO_3_:2Sm^3+^/5.0Ba^2+^ and CaTiO_3_:2Sm^3+^/5.0Bi^3+^ phosphors and 89.88% for CaTiO_3_:2Sm^3+^/5.0Mg^2+^ and CaTiO_3_:2Sm^3+^/0.5Gd^3+^ phosphors. The color purity for the CaTiO_3_:2Sm^3+^/1.0Li^+^ phosphor is 93%. The color purity was also found to improve in the presence of ions of smaller ionic radius (Li^+^/Mg^2+^/Gd^3+^) co-doping and it is maximum for 1.0 mol% Li^+^ co-doping in the CaTiO_3_:2Sm^3+^ phosphor.

We have also calculated the correlated color temperature (CCT) to evaluate the nature of emitted light. The CCT values for the different phosphor samples were evaluated using McCammy's equation as follows:^43^

6 *T* = −449(*n*^3^) + 3525(*n*^2^) − 6823.3(*n*) + 5520.33

where *n* = (*x* − 0.3320)/(*y* − 0.1858) and (*x*, *y*) refer to CIE coordinates. The calculated CCT values were found to be 1975 K, for CaTiO_3_:2Sm^3+^, CaTiO_3_:2Sm^3+^/5.0K^+^, CaTiO_3_:2Sm^3+^/5.0Ba^2+^ and CaTiO_3_:2Sm^3+^/5.0Bi^3+^ phosphors and 1880 K CaTiO_3_:2Sm^3+^/5.0Mg^2+^ and CaTiO_3_:2Sm^3+^/0.5Gd^3+^ phosphors. The CCT value is 1805 K for CaTiO_3_:2Sm^3+^/1.0Li^+^, phosphors. Thus, the CCT values were found to lie in the warm orange-red light region. Therefore, Sm^3+^-doped Sm^3+^/Li^+^/K^+^/Mg^2+^/Ba^2+^/Gd^3+^/Bi^3+^ co-doped CaTiO_3_ phosphor samples were found to be suitable for orange-red lighting devices.

#### 3.2.5. Lifetime measurements

**Fig. 10:**
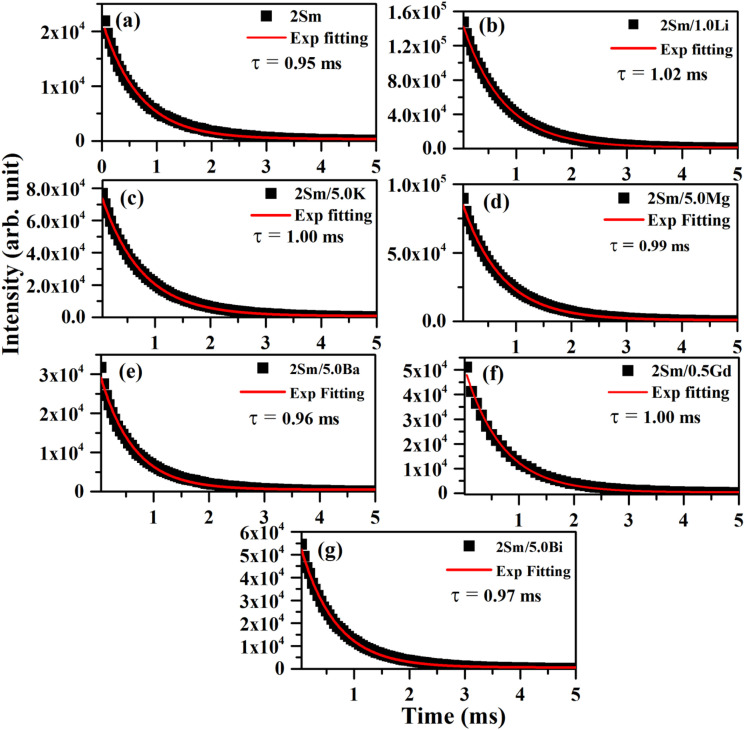
Decay curves for the ^4^G_5/2_ level of Sm^3+^ ions under *λ*_ex_ = 407 nm and *λ*_em_ = 599 nm for (a) CaTiO_3_:2Sm^3+^, (b) CaTiO_3_:2Sm^3+^/1.0Li^+^, (c) CaTiO_3_:2Sm^3+^/5.0K^+^, (d) CaTiO_3_:2Sm^3+^/5.0Mg^2+^, (e) CaTiO_3_:2Sm^3+^/5.0Ba^2+^, (f) CaTiO_3_:2Sm^3+^/0.5Gd^3+^ and (g) CaTiO_3_:2Sm^3+^/5.0Bi^3+^ phosphors.

The lifetime of the ^4^G_5/2_ level of Sm^3+^ ions was measured using the ^4^G_5/2_ → ^6^H_7/2_ transition emitting at 599 nm under 407 nm excitation, and the decay curves are shown in Fig. 10(a)–(g). The decay curves of the ^4^G_5/2_ level of Sm^3+^ ions in different cases were found to fit well using a single exponential relation:^38^

7 *I*(*t*) = *I*_0_ exp(−*t*/*τ*)

where *I*_0_ and *I*(*t*) are the PL emission intensities at time zero and *t* seconds, respectively and ‘*τ*’ is the lifetime.

The value of lifetime for the ^4^G_5/2_ level of Sm^3+^ in the case of CaTiO_3_:2Sm^3+^, CaTiO_3_:2Sm^3+^/1.0Li^+^, CaTiO_3_:2Sm^3+^/5.0K^+^, CaTiO_3_:2Sm^3+^/5.0Mg^2+^, CaTiO_3_:2Sm^3+^/5.0Ba^2+^, CaTiO_3_:2Sm^3+^/0.5Gd^3+^ and CaTiO_3_:2Sm^3+^/5.0Bi^3+^ phosphors were found to be 0.95, 1.02, 1.00, 0.99, 0.96, 1.00 and 0.97 ms, respectively. From this, it is clear that the value of lifetime is increased in the presence of these modifiers (Li^+^/K^+^/Mg^2+^/Ba^2+^/Gd^3+^/Bi^3+^ ions) and it is optimum in the case of Li^+^ ions. The lifetime of the ^4^G_5/2_ level of Sm^3+^ ions follows the trend, *i.e. τ*_Li_ > *τ*_k_ ∼ *τ*_Gd_ > *τ*_Mg_ > *τ*_Bi_ > *τ*_Ba_. The increase in the average crystallite and particle size and the modification in the crystal field around the activator Sm^3+^ ion in the presence of Li^+^/K^+^/Mg^2+^/Ba^2+^/Gd^3+^/Bi^3+^ ions improve the population in ^4^G_5/2_ levels, and therefore, the value of lifetime of ^4^G_5/2_ is increased.^42^ This might be one of the reasons for enhancement in the PL emission intensity.

## 4. Conclusions

Sm^3+^-doped and Sm^3+^/Li^+^/K^+^/Mg^2+^/Ba^2+^/Gd^3+^/Bi^3+^ co-doped CaTiO_3_ phosphors have been synthesized by a solid-state reaction method at 1473 K. The structural and morphological properties of the prepared samples were studied by XRD and SEM measurements. The average crystallite and particle sizes were found to increase in the presence of modifiers and they follow the trend Li^+^ > Mg^2+^ > Gd^3+^ > K^+^ > Bi^3+^ > Ba^2+^. EDX measurements were carried out to verify the elements present in the respective phosphor samples. The infrared measurements of the phosphors showed the presence of Ca–O and Ti–O vibrational bands at 430 and 545 cm^−1^, respectively, indicating that the phosphor has a low phonon frequency. The emission spectra of Sm^3+^ ions showed an intense emission peak at 599 nm due to ^4^G_5/2_ → ^6^H_7/2_ transition upon excitation at 407 nm wavelength and the emission intensity is maximum for 2 mol% of Sm^3+^ ion. The emission intensity is quenched for a higher concentration of Sm^3+^ ions. Therefore, the surface modifiers were used to further enhance the emission intensity. It was found that co-doping 1.0 mol% Li^+^, 5.0 mol% K^+^, 5.0 mol% Mg^2+^, 5.0 mol% Ba^2+^, 0.5 mol% Gd^3+^ and 5.0 mol% Bi^3+^ ions enhances the emission intensity by 6.3, 4.0, 5.1, 1.4, 5.0 and 2.5 times respectively as compared to the 2Sm^3+^-doped CaTiO_3_ phosphor. The increase in emission intensity is due to the modification of the crystal field around the Sm^3+^ ions in the CaTiO_3_ host as well as the increase in average crystallite and particle sizes in the presence of these ions. It was found that the size (ionic radii) and charge compensation of the ion together play a dominant role. The enhancement is more after co-doping with smaller radius ions (Li^+^, Mg^2+^ and Gd^3+^), among which Li^+^ shows the largest enhancement. This is because ions of smaller size will be able to go closer to the activator ion and the charge imbalance causes a larger field. The emission intensity is maximum in the case of the CaTiO_3_:2Sm^3+^/1.0Li^+^ phosphor due to its smaller ionic radii and used as a charge compensator as well as it has the largest average crystallite and particle size. The CIE color coordinates and correlated color temperature (CCT) showed the orange-red color with a color purity as high as 93% in the case of the CaTiO_3_:2Sm^3+^/1.0Li^+^ phosphor. The lifetime of the ^4^G_5/2_ level of Sm^3+^ ions was also found enhanced in the presence of Li^+^/K^+^/Mg^2+^/Ba^2+^/Gd^3+^/Bi^3+^ ions and it follows the trend *τ*_Li_ > *τ*_k_ ∼ *τ*_Gd_ > *τ*_Mg_ > *τ*_Bi_ > *τ*_Ba_. From these studies, it is suggested that Sm^3+^-doped and Sm^3+^/Li^+^/K^+^/Mg^2+^/Ba^2+^/Gd^3+^/Bi^3+^ co-doped CaTiO_3_ samples may be suitable for display devices and for LEDs under n-UV excitation.

## Conflicts of interest

This work does not have any conflict of interest.

## Supplementary Material
